# Suppression of PKC causes oncogenic stress for triggering apoptosis in cancer cells

**DOI:** 10.18632/oncotarget.16047

**Published:** 2017-03-09

**Authors:** Suthakar Ganapathy, Bo Peng, Ling Shen, Tianqi Yu, Jean Lafontant, Ping Li, Rui Xiong, Alexandros Makriyannis, Changyan Chen

**Affiliations:** ^1^ Center for Drug Discovery, Northeastern University, Boston, MA, USA; ^2^ The First Affiliated Hospital, Zhengzhou University, Zhengzhou, China; ^3^ The Institute of Clinic Sciences, Sahlgrenska Academy, Gothenburg, Sweden

**Keywords:** apoptosis, Ras, Akt, ROS, p73

## Abstract

Gain of functional mutations in *ras* occurs in more than 30% of human malignancies and in particular 90% of pancreatic cancer. Mutant *ras*, via activating multiple effector pathways, not only promote cell growth or survival, but also apoptosis, depending upon cell types or circumstances. In order to further study the mechanisms of apoptosis induced by oncogenic *ras*, we employed the *ras* loop mutant genes and demonstrated that Akt functioned downstream of Ras in human pancreatic cancer or HPNE cells ectopically expressing mutated *K-ras* for the induction of apoptosis after the concurrent suppression of PKC α and β. In this apoptotic process, the redox machinery was aberrantly switched on in the pancreatic cancer cells as well as prostate cancer DU145 cells. p73 was phosphorylated and translocated to the nucleus, accompanied with UPR activation and induction of apoptosis. The *in vitro* results were corroborated by the *in vivo* data. Thus, our study indicated that PKC α and β appeared coping with oncogenic Ras or mutated Akt to maintain the balance of the homeostasis in cancer cells. Once these PKC isoforms were suppressed, the redox state in the cancer cells was disrupted, which elicited persistent oncogenic stress and subsequent apoptotic crisis.

## INTRODUCTION

Ras proteins exist in two conformations: a GTP-bound active state and GDP-bound inactive state. The ratio of GTP and GDP bound to cellular Ras proteins is controlled by guanine nucleotide exchange proteins and GTPase-activating proteins, the enzymatic activity of which responds to extracellular stimuli (such as growth factors) [1–4]. Once activated, GTP-bound Ras interacts with target enzymes/effectors and triggers kinase cascades to regulate various cellular activities. Ras, through controlling the activity of these effectors, is able to influence cell behaviors. The best characterized effectors of Ras are various protein serine/threonine kinases, for example, Raf/MEK/MAPK, PI3K/Akt or Rho/Ral [2, 5]. Studies also demonstrated that Ras-governed pathways could induce apoptosis in several types of cancer cells [6–8]. In response to different apoptotic stimuli, Ras differentially used its downstream effector pathways and re-directed the cells to apoptosis. PI3K/Akt pathway was shown to be able to upregulate several ROS-related enzymes (such as NADPH oxidase), and play important roles in the regulation of endoplasmic reticulum (ER)-stress induced apoptosis [9, 10].

The uses of the high-throughput RNA-interference (siRNA) technique to selectively inhibit gene expressions, together with the knowledge of the full sequence of the genome, have made possible to identify intracellular targets of cancer cells, through large-scale functional genomic screens [11, 12]. Cancer cells often develop secondary dependencies on genes/factors for survival. Perturbation of these genes/factors results in oncogene-specific synthetic lethality. Such lethality often involves genes/factors within the same or parallel pathways that are required for an essentially vital function. Using siRNA screening, it has uncovered kinase-specific vulnerabilities in tumors harboring mutated *ras* [11, 12]. Furthermore, studies (including ours) demonstrated that loss of PKC (in particular the phorbol ester-dependent subgroup of PKC isoforms), together with Ras mutations, were synthetically lethal [13–17]. Since about 30–40% of human tumors contain an oncogenic *ras* and attempts to directly target mutated Ras proteins with small molecular weight inhibitors have proved to be unsuccessful [18], the research focus is now on identifying targeting pathways that function downstream of or parallel with oncogenic Ras. The inhibition of one of these pathways would specifically trigger apoptosis in the tumor cells harboring mutant *ras*, but be less or minimal toxic to surrounding normal tissues/cells.

Reactive oxygen species (ROS) are a diverse group of reactive, short-lived, oxygen containing species (such as superoxide radical, hydrogen peroxide and hydroxyl radical), and constantly generated in cells during normal aerobic metabolism [19–22]. Physiological levels of ROS are required for cell signaling cascades. The consequences of aberrant ROS increases in cells cause permanent structural changes in DNA (such as DNA strand breaks), initiation of lipid peroxidation, alterations of intracellular signaling pathways (such as ER stress-related proteins) or induction of apoptosis [23–25]. Cells possess an array of defense systems to suppress and scavenge excess ROS as well as repair related damages, in order to protect from being injured. When the defense systems cannot effectively suppress aberrant increases of ROS, a persistent oxidative or ER stress is triggered. Such stresses often cause the activation of the unfolded protein response (UPR) and in some cases, apoptosis is induced [26–28]. The proper balance of intracellular redox state is crucial for cell survival. Under normal growth conditions, a fraction of oxygen is converted to superoxide by NADH dehydrogenase and other enzymes of the mitochondrial respiratory chain [29, 30]. Superoxide is then converted to H_2_O_2_ by superoxide dismutase (SOD) [29, 30]. H_2_O_2_ at normal levels is able to diffuse through cell membranes and needed for regulating various cellular activities [31–33]. Persistently high amounts of ROS often cause damages in the mitochondrial transmembrane potential or activate UPR, which thereby could trigger cell death [26–28]. ROS-induced apoptosis was indicated to be via the activation of PI3K/Akt, p53 or other factors [15, 34, 35]. However, the mechanisms that control cell fate: cell survival versus death in response to oxidative or ER stress, have not been fully investigated.

The ER is a cytosolic compartment where proteins and lipids are synthesized. Chaperones resided in the ER facilitate proper protein folding, maintain proteins in a folded structure or prevent protein folding intermediates from aggregating [36–38]. BIP is one of the best characterized ER chaperone proteins and its synthesis can be stimulated by various stresses (including oxidative stress) that sometimes disrupt ER function and perturb homeostasis [39–41]. Studies suggested that increases in BIP expression are the indicator of ER stress. The ER compartment is very flexible and can accumulate partially folded proteins. Once accumulations of improper folded proteins exceed the limit of the ER, the UPR is activated, leading to diseases or apoptosis [42–45].

In the present study, using different *ras* loop mutant constructs, we intended to identify which Ras effector(s), together with suppression of PKC, was/were synthetically lethal in the cancer cells. The results suggested that Akt acted downstream of oncogenic Ras in the cancer cells and induced apoptosis after co-suppressing PKC α and β.

## RESULTS

### Pancreatic cancer cells expressing mu-K-ras underwent apoptosis after the co-suppression of PKC α and β

**Figure 1:**
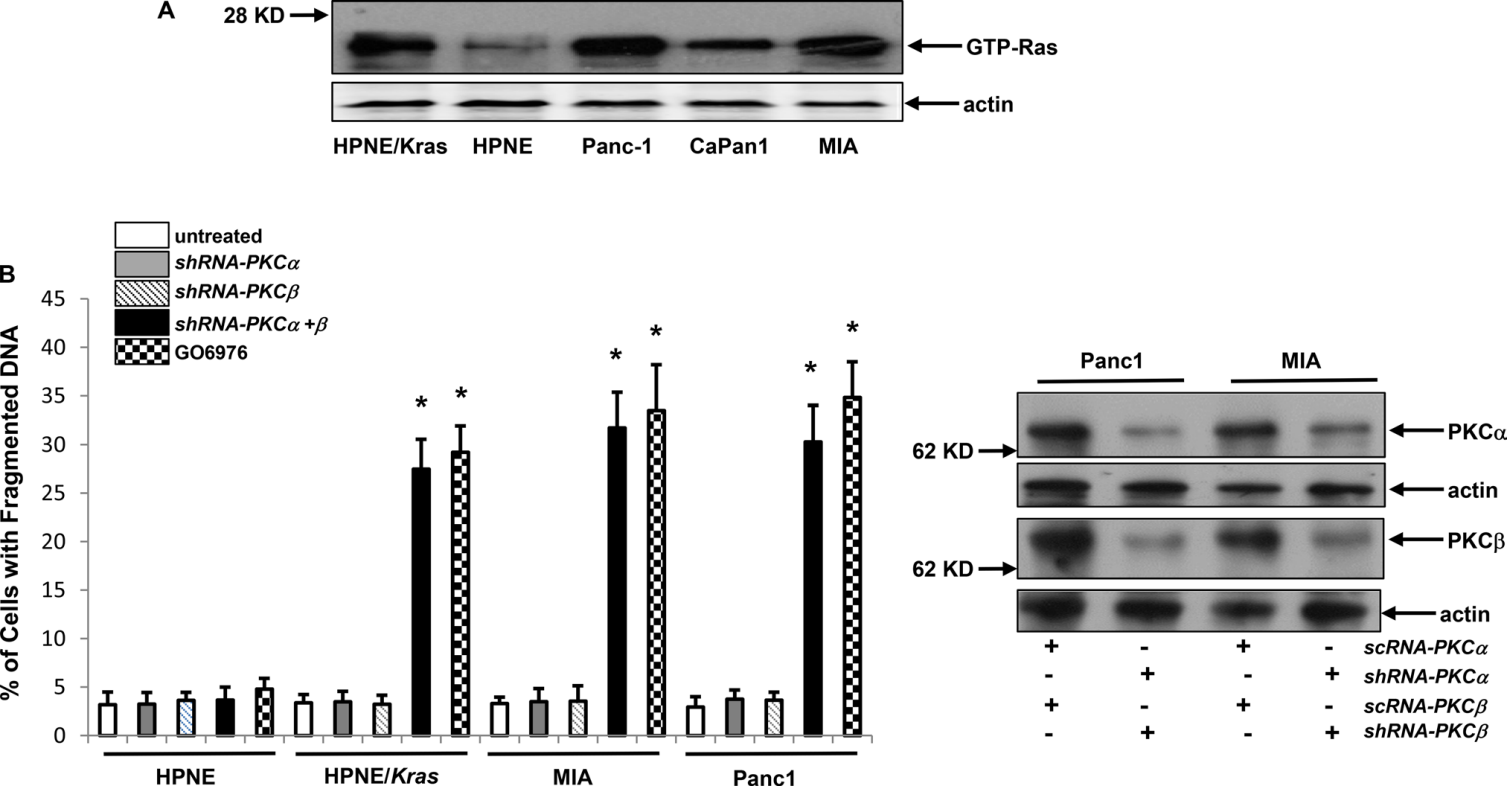
Mutant *K-ras* and loss of PKC α and β were synthetically lethal (**A**) Ras GTP-binding activity was analyzed in the cells using active Ras pull-down and detection kit. Evenly loadings of the lysates were normalized by actin expression. (**B**) Cells were infected with *sc* or *shRNA-PKC α* or *β* for 48 h and the expressions of corresponding proteins were then analyzed by immunoblotting (right panels). After the infection of *shRNA-PKC α*, *β* or both as well as treatment of GO6976 (10 uM) for 48 h, DNA fragmentation assay was conducted to detect the induction of apoptosis in the cells (left panel). The error bars are SD from 5 independent experiments (*n* = 5, *ρ* < 0.05).

The inhibition of PKC triggered apoptosis in cultured cells expressing *v-ras* or cancer cells harboring mutated *ras* [13–17]. However, the underlying mechanisms by which oncogenic *ras* induces apoptosis remain unclear. For this reason, human pancreatic epithelial cells (HPNE) or its HPNE stably transfected with *mu-K-ras* as well as human pancreatic cell lines Panc-1 or MIA harboring mutated *K-ras* were used to test their sensitivities to PKC inhibition for the induction of apoptosis. Ras activation status in these cells was first analyzed using Active Ras Pull-Down and Detection kit (Figure 1A). The increased amount of the GTP-bound Ras was detected in all cells expressing aberrant *K-ras*, in comparison that only the baseline level of active Ras was revealed by the antibody in HPNE cells. Our previous reports demonstrated that murine fibroblasts ectopically expressing mutant *ras* were susceptible to apoptosis after the co-suppression of PKC α and β isoforms [13–17]. Therefore, the susceptibility of the pancreatic cancer cells to the co-inhibition of PKC α/β was studied. The effect of the knockdown of PKC α or β by the *shRNAs* was analyzed by immunoblotting. The *shRNA*s, but not the *scRNA*s, efficiently knocked down these two PKC isoforms (Figure 1B, right panels). The DNA fragmentation assay was then conducted (Figure 1B, left panel). Approximately 30% of HPNE/*K-ras*, Panc1 and MIA cells, but not HPNE cells, underwent apoptosis after the knockdown of PKC α/β.

### ROS is upregulated in response to the co-suppression of PKC α and β

**Figure 2:**
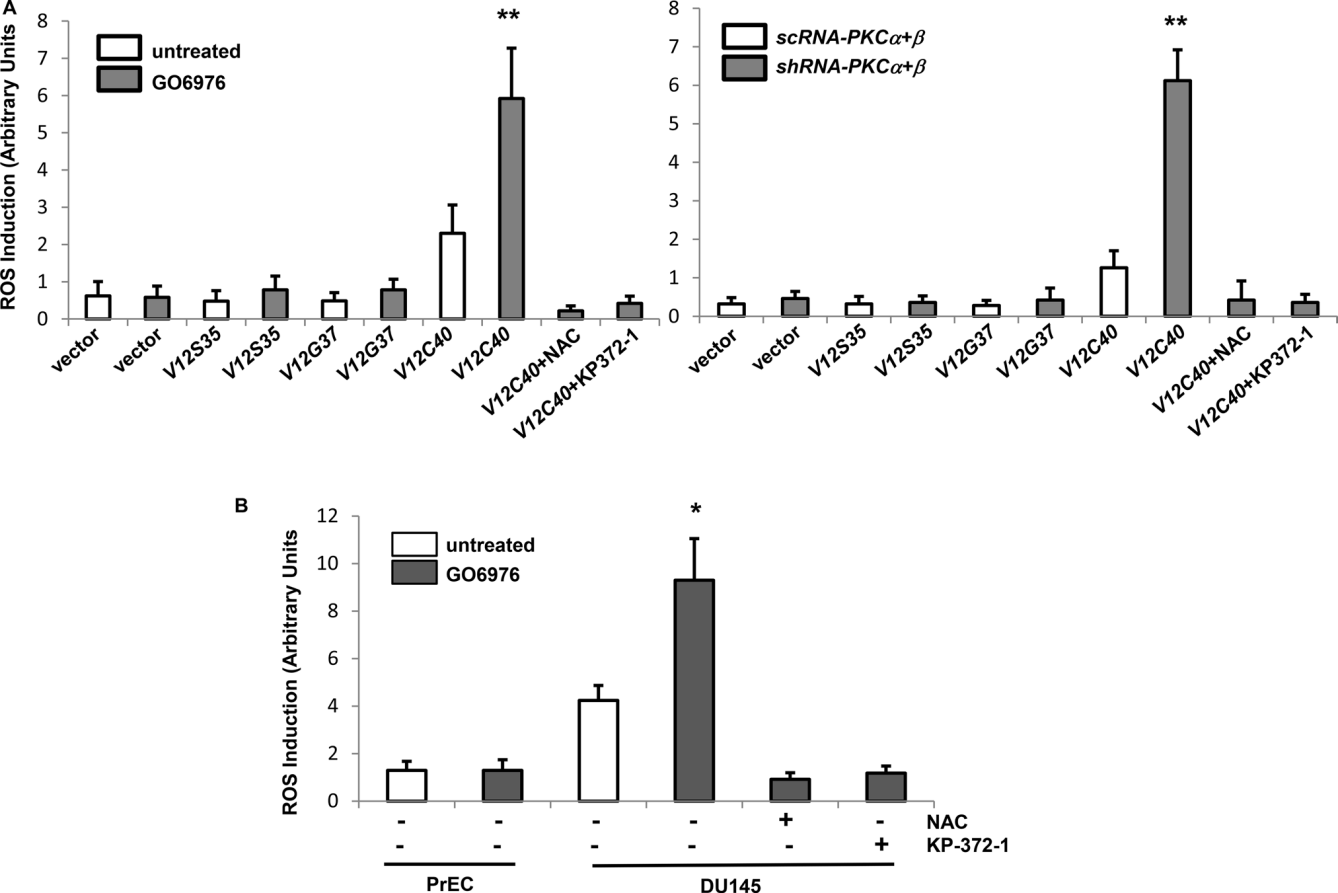
ROS was upregulated via PI3K/Akt pathway after the co-suppression of PKC α and β (**A**) HPNE cells were stably transfected with the *ras* effector loop mutant constructs. After being treated with GO6976 or co-infected with *sc* or *shRNA-PKC α* and *β*, the amounts of ROS in the cells were measured. The error bars are SD from 5 independent experiments (*n* = 5, *ρ* < 0.005). (**B**) Prostate epithilieal PrEC and cancer DU145 cells were treated with NAC or KP-372-1 prior to GO6976 treatment, and the levels of ROS were measured. The error bars are SD from 5 independent experiments (*n* = 5, *ρ* < 0.05).

ROS is required for various cellular activities. However, aberrant increases of ROS triggers oxidative stress, sometimes resulted in apoptosis [19–24]. To further test if the co-suppression of PKC α and β perturbed the redox state, *ras* effector loop mutant constructs were employed. *V12S35* (the protein product of which is able to bind to and activate Raf), *V12C40* (the protein product of which preferentially activates PI3K/Akt), or *V12G37* (encoded protein interacts with RalGDS) was introduced into HPNE cells, respectively and the protein expressions of the Ras mutants were confirmed by immunoblotting (data not shown) [25]. The levels of ROS in response to co-knockdown of PKC α/β or treatment of GO6976 were measured in HPNE cells with or without overexpressing *v-Kras* loop mutant constructs (Figure 2A). The introduction of *V12C40* mutant gene caused a slightly increased level of ROS in HPNE cells, which was significantly upregulated by the addition of GO6976 (Figure 2A, left panel) or co-knockdown of PKC α/β (Figure 2A, right panel). Such upregulation of ROS was suppressed by N-acetyl-L-cysteine (NAC, a ROS inhibitor) (data not shown). In comparison, other ras loop mutants did not cause the increases of ROS in the cells, even in the absence of PKC. To confirm the role of PI3K/Akt, human prostate epithelial PrEC and cancer DU145 cells that express active *Akt* were assayed for ROS accumulation in our experimental setting (Figure 2B). The amount of ROS in untreated DU145 cells was slight elevated, but significantly upregulated by GO6976 treatment, which was suppressed by NAC or KP-372-1 (an Akt inhibitor). GO6976 did not affect ROS level in PrEC cells. The data suggested a linear relationship between oncogenic Ras and PI3K/Akt pathway in the perturbation of the balance of the redox state in the cells after the co-inhibition of PKC α and β.

### Oxidative regulators are activated in a PI3K/Akt-dependent fashion

**Figure 3:**
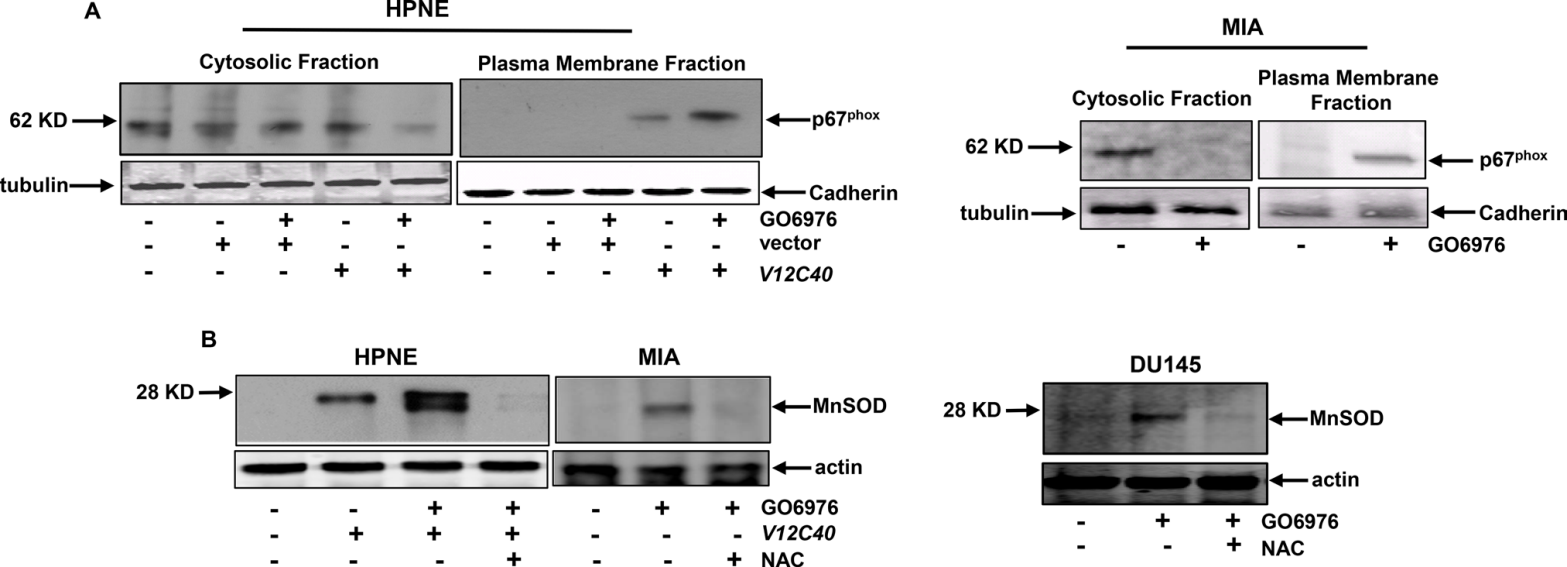
ROS regulators were activated after co-suppression of PKC α and β (**A**) After the treatments, the cytosolic or plasma membrane fraction from the cells were isolated and immunoblotted for p67PHOX expression. The evenly loadings were normalized by tubulin or cadherin. (**B**) MnSOD expression in the cells after the treatments was analyzed by immunoblotting.

NADPH oxidase is a membrane-bound enzymatic complex and one of the sources of intracellular superoxides [19–22]. Its cytosolic components (p67^phox^, p47^phox^ and p40^phox^) translocated to the plasma membrane in response to oxidative stress [19–22]. Therefore, HPNE cells with or without transiently expressing *V12C40* (Figure 3A, left panels) as well as pancreatic cancer MIA cells (Figure 3A, right panels) were treated with GO6976. The plasma membrane or cytosolic fractions were then isolated, and immunoblotted with the antibody for p67^phox^ expression. p67^phox^ was present in the cytosolic fraction of HPNE cells expressing vector alone regardless of GO6976 treatment. In comparison, a high amount of p67^phox^ was present in the cytosolic fraction of untreated HPNE/*V12C40* cells. After GO6976 treatment, a significant increased amount of p67^phox^ appeared in the plasma membrane fraction of HPNE/*V12C40* cells. The similar pattern of p67^phox^ plasma membrane translocation was observed in GO6976-treated MIA cells. The expression of MnSOD (Mn^2+^ superoxide dismutase, one of ROS modulators) in HPNE, MIA and DU145 cells was then analyzed by immunoblotting (Figure 3B). A slight increase of MnSOD in untreated HPNE/*V12C40* cells was detected. After GO6976 treatment, MnSOD expression was dramatically upregulated, which was blocked by the addition of NAC, indicating the role of ROS in the induction of MnSOD. The expression of another ROS regulator HO-1 was also examined under the same experimental conditions, and similar results were obtained (data not shown). Overall, the data further indicated that the co-suppression of PKC α/β perturbed the redox machinery.

### p73 was activated in an Akt-dependent fashion after co-suppressing PKC α and β

**Figure 4:**
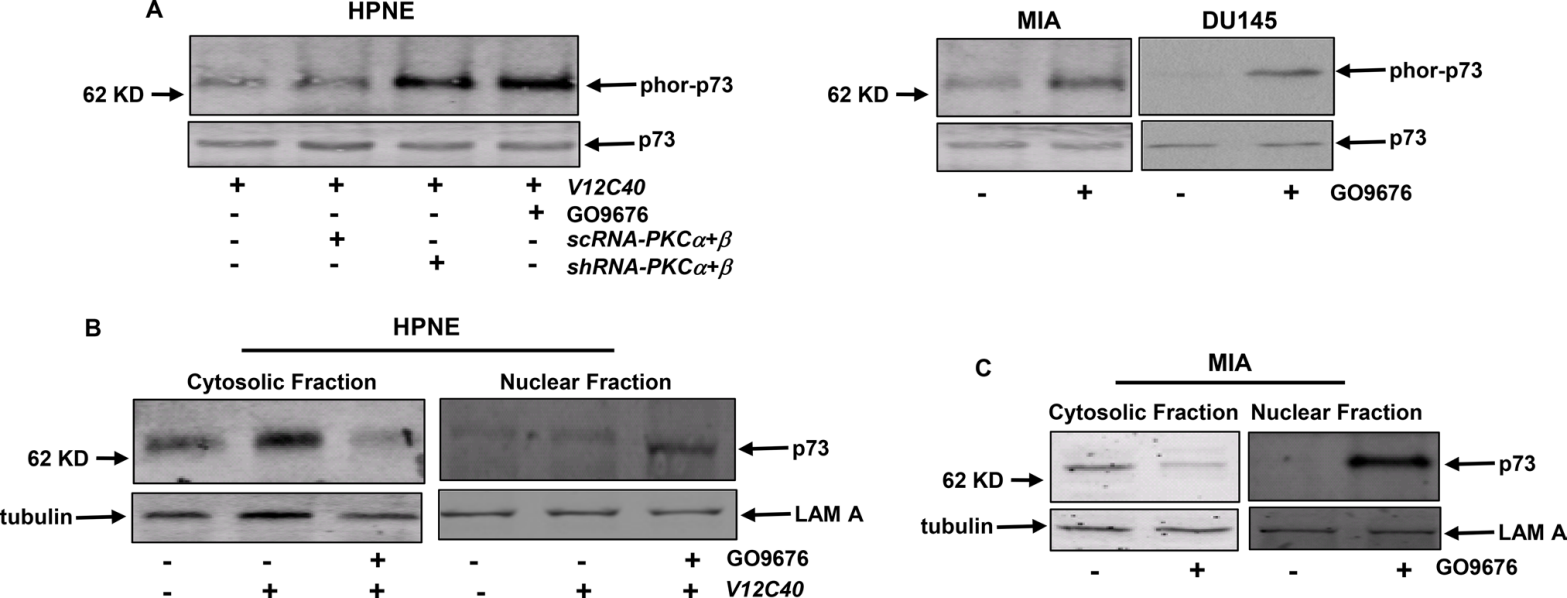
p73 is phosphorylated and translocated to the nucleus in response to GO6976 treatment (**A**) HPNE cells ectopically expressing *V12C40* as well as pancreatic cancer MIA or DU145 cells were either co-infected with *shRNA-PKC α/β* or treated with GO6976. Subsequently, lysates were immunoprecipitated with anti-p73 antibody and then immunoblotted with the antibody recognizing the phosphorylated p73. The evenly loadings of the samples were normalized by re-probing the blot with anti-p73 antibody. (**B** and **C**) After the treatments, the cytosolic or nuclear fractions were isolated and immunoblotted for p73 expression. The evenly loadings were normalized by tubulin or Lam A.

p73 is a member of p53 tumor suppressor family and involved in the regulation of apoptosis upon genotoxic stress or exposure to apoptotic stimuli [16]. It was reported that active p73 could re-locate to the nucleus and participate the induction of apoptosis there [46, 16]. Therefore, p73 function in our experimental setting was examined. After GO6976 treatment, cell lysates from HPNE cells with or without overexpressing *V12C40*, and from MIA or DU145 cells were prepared to test the expression of phor-p73 (Figure 4A). The phosphorylated form of p73 was detected in HPNE/*V12C40*, MIA or DU145 cells only after the co-suppression of PKC α and β. Subsequently, the subcellular localization of p73 in our experimental setting was analyzed (Figure 4B). The cytosol and nuclear fractions were isolated from HPNE or NPNE/*V12C40* cells with or without GO6976 treatment and immunoblotted with anti-p73 antibody. This tumor suppressor was detected in the cytosol fractions of untreated HPNE and HPNE/*V12C40* cells, which was absent in the nuclear fractions. After GO6976 treatment, p73 was disappeared in the cytosolic fraction of HPME/*V12C40* cells, and appeared in its nuclear fraction. The similar pattern of the nuclear translocation of p73 was observed in GO6976-treated MIA cells (Figure 4C). The data implicated that p73 was activated and perhaps played an indispensable role in this apoptotic process.

**Figure 5:**
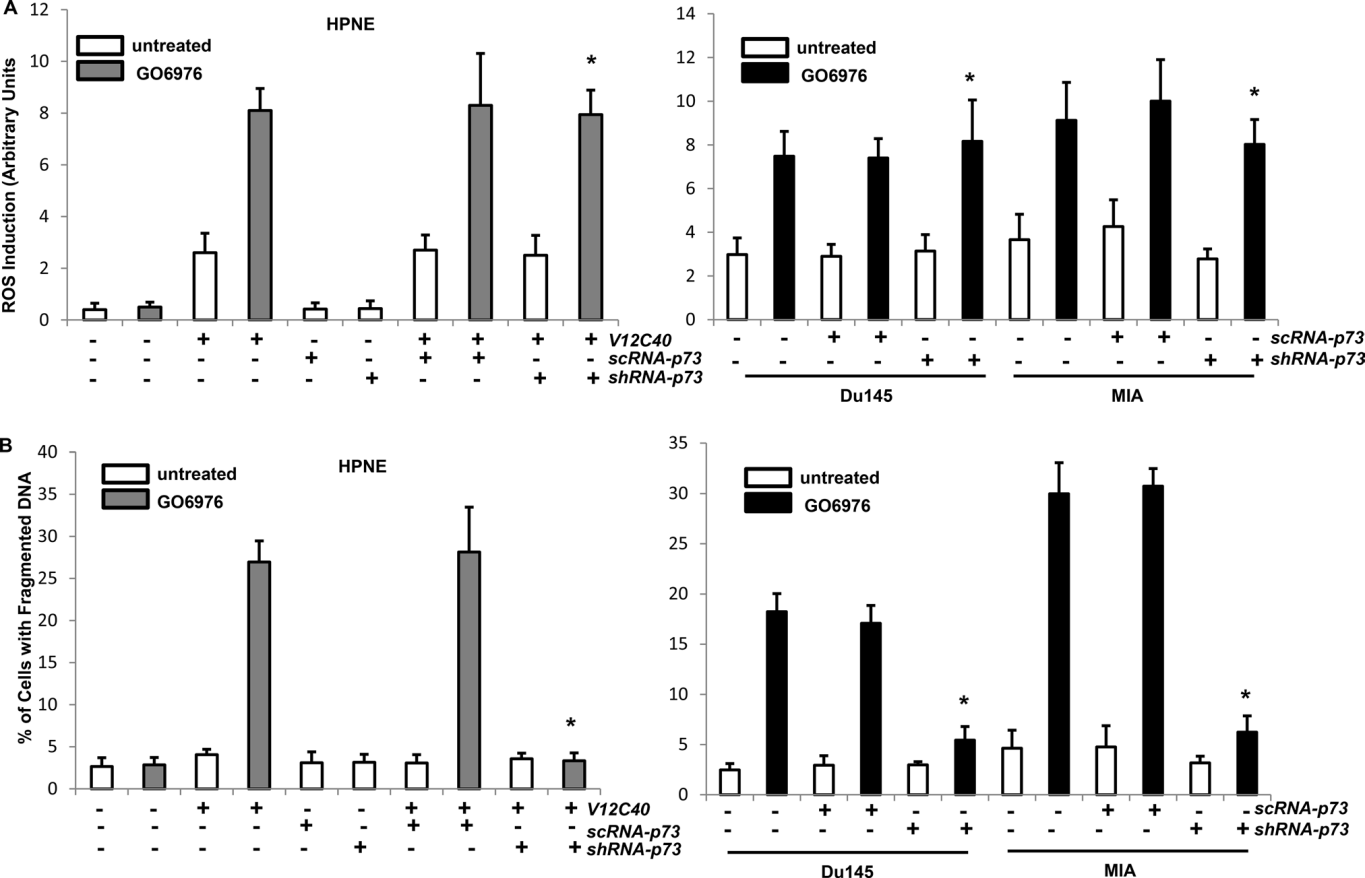
Induction of ROS and apoptosis in the absence of p73 (**A**) After infected with *sc* or *shRNA-p73*, cells were treated with NAC or KP-372-1 prior to GO6976 treatment, and the levels of ROS were then measured. The error bars are SD from 5 independent experiments (*n* = 5, *ρ* < 0.05). (**B**) After the treatments, DNA fragmentation assay was conducted to detect the induction of apoptosis in the cells. The error bars are SD from 5 independent experiments (*n* = 5, *ρ* < 0.01).

To further test if both the upregulation of ROS and induction of apoptosis occurred in our experimental setting were p73-dependent, the level of ROS (Figure 5A) and onset of apoptosis (Figure 5B) were analyzed. Again, a slight increase of ROS was revealed in untreated HPNE/*V12C40* cells. The knockdown of *p73* did not affect the upregulation of ROS mediated by GO6976 treatment. The similar results were obtained from GO6976-treated pancreatic or prostate cancer cells after p73 was knockdown. Subsequently, the induction of apoptosis in the cells was analyzed after knockdown of *p73*. In the absence of *p73*, HPNE/*V12C40*, MIA and DU145 cells were no longer sensitive to GO6976 treatment for the induction of apoptosis. The data suggested that the perturbation of ROS in our experimental setting occurred upstream of p73 and, the induction of apoptosis was dependent upon p73.

### UPR was activated after the co-suppression of PKC α and β

**Figure 6:**
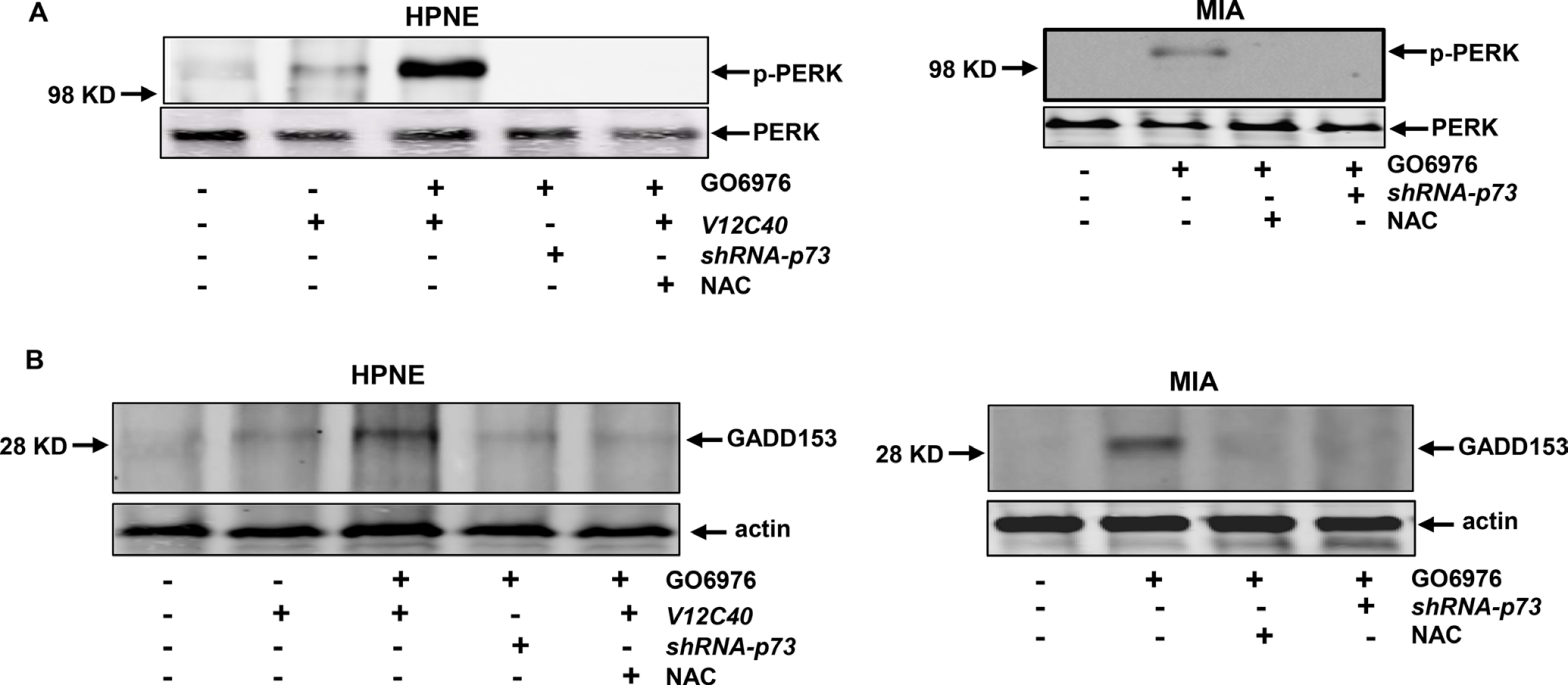
ER stress and UPR activation were triggered in the cells in response to GO6976 treatment (**A**) In the presence or absence of p73, the phosphorylated PERK in HPNE, HPNE/*V12C40* or MIA cells was analyzed by immunoblotting. (**B**) GADD153 expression was analyzed after the treatments as described above.

The ER plays an important role in sensing cellular insults, persistent of which often activates the unfolded protein response (UPR) [36–38]. PERK is one of UPR sensors and activated or phosphorylated during persistent ER stress. To test whether the UPR was activated in our experimental setting, the expression of the phosphorylated form of PERK in the cells after the treatment of GO6976 was analyzed (Figure 6A). The phosphorylated PERK was detected in GO6976-treated HPNE/*V12C40* or MIA cells, which was blocked by the addition of NAC or infection of *shRNA-p73*. PERK was not phosphorylated in untreated cells, as expected. GADD153 is an apoptotic factor and often induced in UPR-mediated apoptosis [36–38]. Immunoblotting analysis was performed to test GADD153 expression (Figure 6B). GADD153 was almost undetectable in untreated cells. After treated with GO6976, this apoptotic factor was induced in HPNE/*V12C40* and MIA cells, which was suppressed by the addition of NAC or by knockdown of p73. It appeared that apoptosis triggered by the co-suppression of PKC α and β was the consequence of UPR activation, in which ROS and p73 were required.

### Induction of apoptosis occurs in xenografted pancreatic tumors

**Figure 7:**
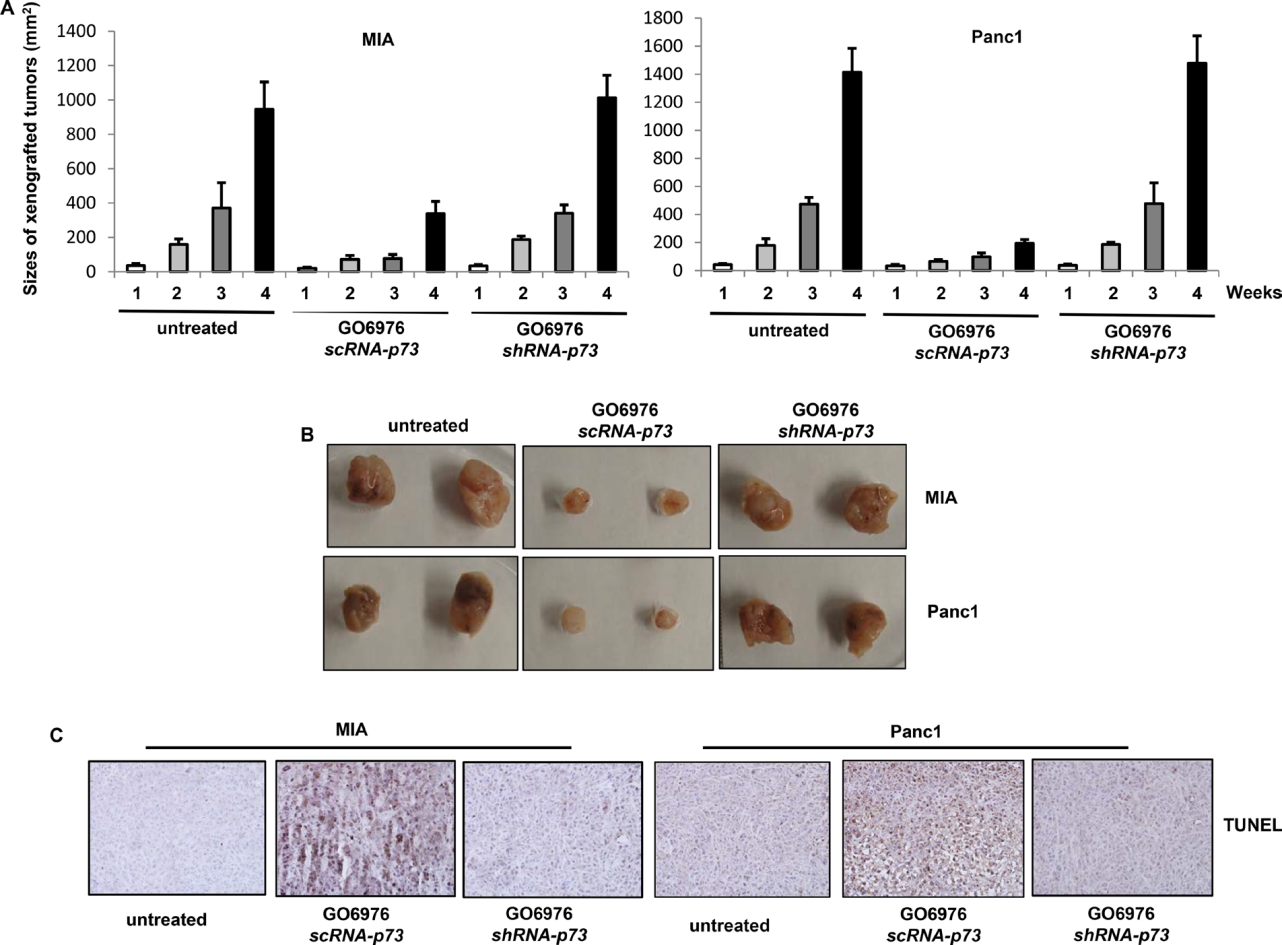
Induction of apoptosis in the xenografted tumors (**A**) Cells were inoculated subcutaneously into the nude mice. GO6976 was injected peritoneally after the inoculation and subsequently administrated every 3 days. One week later, the diameters of the tumors were measured and then every week for consecutive 4 weeks. The error bars represent the SD (*n* = 4, *p < 0.05). (**B**) After the tumors were dissected from the mice, the pictures of the tumors were taken. (**C**) The slides mounted with the tumor samples were stained with TUNEL reagent.

To further confirm the results from the *in vitro* experiments, xenograft assay was performed (Figure 7). After the inoculation of human pancreatic cancer MIA or Panc1 cells without or with stably expressing *scRNA-p73* or *shRNA-p73* into nude mice, respectively, GO6976 (30 mg/kg) was injected into the mice intraperitoneally, which was given every 3 days. One week after the inoculation when the tumors became detectable, the diameters of the tumors were measure every week for consecutive 4 weeks (Figure 7A). The xenografted tumors were formed in untreated mice inoculated with MIA or Panc1 cells. In comparison, the xenografted MIA or Panc1 tumors expressing *scRNA-p73* in the mice grew very slow after received routine GO6976 injection. After the knockdown of *p73*, GO6976 injection could not block the tumor growth in the mice. The pictures of the tumors with or without the knockdown of *p73* were taken (Figure 7B). The slides mounted with the tumor samples were stained with TUNEL reagent (Figure 7C). TUNEL staining was negative in untreated or GO6976-injected tumors after p73 was knockdown. However, the samples from GO6976-injected tumor cells transfected with *scRNA-p73* were strongly stained with TUNEL reagent. The xenograft assay was also conducted with DU145 cells with or without the knockdown of *p73*. The similar data were obtained (data not shown). The *in vivo* data corroborated well with those obtained from the *in vitro* experiments. The data further suggested that the co-suppression of PKC α and β sensitized cancer cells harboring oncogenic *ras*, which is dependent upon p73.

## DISCUSSION

Mutated *ras* or aberrant *ras* effectors are often present in various types of human malignancies. For example, in more than 90% of pancreatic cancer patients, mutated *K-ras* is detected, and active *Akt* are often seen in refractory prostate cancer among other types of cancers. [47]. Lack of effective treatments for these devastating diseases is the big challenge. Therefore, the objective of our current study was to search for new targets, which might help developing effective therapeutic strategies against cancers harboring aberrant *ras*. Using the *ras* loop mutant constructs, we demonstrated that PI3K/Akt signaling appeared functioning downstream of mutant Ras and was able to activate cell death program after the co-suppression of PKC α and β. In this apoptotic process, ROS was highly increased, resulted in ER stress and UPR activation for the induction of apoptosis. Furthermore, p73 was phosphorylated and translocated from the cytosol to the nucleus. However, p73 appeared downstream of ROS and played an important role in the initiation of apoptosis. The animal experiments corroborated well with the data from the *in vitro* studies. The study suggested that mutated *ras*, together with loss of PKC α and β, are incompatible for the viability of pancreatic or refractory prostate cancer cells. It also indicated that tumor suppressor p73 is a key element for triggering apoptosis in the cancer cells.

The mutational activation of *ras* is one of the key events during the initiation and development of various types of human malignancies, especially pancreatic cancer [1–5]. In the process of the malignant transformation, oncogenic Ras interacts and further switches on various downstream effector pathways, which triggers phosphorylation chain reactions and activates transcriptional factors. Despite the essential involvement in cell growth and differentiation, hyper-active Ras was shown to be able to induce apoptosis when some of its effector or supporting factors are suppressed [13–17]. For example, the treatment of PKC inhibitors is able to sensitize transformed cells ectopically expressing oncogenic *ras* or human cancers harboring mutant *K-ras* to apoptosis [48, 49]. In current study, we further demonstrated the underlying mechanisms by which the co-suppression of PKC α and β appeared essential for the viability of pancreatic cancer cells harboring mutated *K-ras* or prostate cancer cells expressing active *Akt*. The data suggested that these two PKC isoforms coped with aberrant Ras or Akt to keep the homeostasis in the cancer cells. Once these two isoforms were knocked out or inhibited, the oncogenes could not maintain malignant metabolic needs and fatal crises occurred. Thus, PKC α and β isoforms seem potentially therapeutic targets for developing new strategies to treat cancers harboring aberrant *ras* or *Akt*.

Levels of ROS are often altered in response to mitogenic stimuli. Persistent increases of ROS trigger the structural changes of the cytoskeleton, which often promote cell transformation or tumorigenesis [19–22]. Upon Epithelial growth factor (EGF) stimulation, ROS production was increased in the cells overexpressing oncogenic *ras*, which caused cancer progression [50, 51]. Oncogenic stresses induced by *myc* or *ras* oncogene often perturbed the state of intracellular redox, which led to chromosomal aberrations and disruption of genetic integrity for tumor promotion. Also, high increases of ROS were shown to play an obligatory role in the induction of apoptosis [14, 52]. For example, in TNFα-induced cell death, NF-κB and JNK cooperated to mobilize ROS signaling and further trigger caspase cascade [14, 52]. Ectopic expression of oncogenic *ras* in murine fibroblasts activated cell death program via inducing ER stress and UPR [14, 15]. In this study, we demonstrated that the concurrent inhibition of PKC α and β caused a significant increase of ROS that disrupted the equilibrium of the redox state in cancer cells harboring mutant *K-ras* or active *Akt*. The study indicated the important roles of PKC α and β in maintaining the homeostasis of these cancer cells.

PKC family consists of multiple serine/threonine kinase isoforms, some of which are structurally distinct or functionally diverse. The roles of some of PKC isoforms in the regulation of cell growth or death are rather controversial, depending upon cell types or cellular contexts. For example, PKC α, β, δ possessed dual roles in the regulation of cancer development or programmed cell death [16, 17, 48], which reflect the complexity of these isozymes. Cross-talks among PKC isoforms or with other intracellular signal transducers were often occurred in different subcellular compartments, which guided cells to participate different cellular activities. The interconnection of PKC and Ras signaling pathways was demonstrated in lymphocytes [53]. Upon mitogenic stimulations, the SH2 binding sites of PKC in T lymphocytes were shown to be phosphorylated and then recruited other signal transducers (such as Ras) via Grb2/SOS for forming the complexes, resulting in the activation of Ras signaling [53]. Using GO6976 that specifically inhibits PKC α and β isoforms or *shRNAs-PKC α/β*, we showed that the pancreatic cancer cells harboring mutant *K-ras* or prostate cancer cells expressing active *Akt* are being efficiently sensitized to apoptosis, which again suggested the cooperation of these pathways.

As a p53 family member, p73 shares the structural and functional homologue with p53 [54, 55]. This tumor suppressor plays an important role in DNA damage- or stress-induced apoptosis. The nuclear c-Abl was shown to be activated by genotoxic stress and further phosphorylated p73 for the induction of apoptosis [46, 55]. In this process, active p73 translocated to the nucleus, and interacted with c-Abl to initiate caspase cascade. In response to ionizing radiation or cisplatin treatment, p73 played a crucial role in initiating the apoptotic signaling [56, 57]. In our current experimental setting, p73 was phosphorylated and translocated from the cytosol to the nucleus for the induction of apoptosis. However, p73 activity is dispensable for the upregulation of ROS, but not for triggering UPR activation. The underlying mechanisms by which p73 elicits the sensors of UPR remain to be further investigated.

In summary, mutations in *K-ras* or *Akt* are uniquely detected in many types of human cancers. Thus, there is an eminent need for identifying intracellular targets to develop new strategies to treat these cancers. Our study demonstrated an apoptotic signaling pathway, in which *mu-K-Ras* utilized its downstream PI3K/Akt pathway to induce apoptosis in the absence of PKC α and β. In this apoptotic process, ROS was upregulated, accompanied with the activation of UPR and p73. The results indicated that PKC PKC α and β are important factors for the viability of cancer cells harboring mutant K-Ras or active Akt. Therefore, our investigation provides further information about the underlying mechanisms of apoptosis elicited by mutant K-Ras. Such knowledge may be exploited therapeutically for treating malignancies, especially pancreatic cancer or refractory prostate cancer.

## MATERIALS AND METHODS

### Cells and reagents

Human pancreatic epithelial HPNE, its stable *mu-Kras* transfectant HPNE/*Kras*, pancreatic cancer MIA, Panc1 and prostate cancer DU145 cells were purchased from ATCC (Manassas, VA) and the details of the phenotypes of the cells were provided by ATCC. The cells were frozen and kept in a -150^0^ C freezer after purchasing so that the authentications of the cells are preserved. HPNE cells were cultured in M3 medium containing 10% heat-inactivated fetal bovine serum and epithelial growth factor (EGF), penicillin and streptomycin (Invitrogen, CA). HPNE/*Kras* cells were maintained in the same M3 medium with 150 ug/ml of purimycin to keep the transfection pressure. Other cell lines were grown in Dulbecco's Modified Eagles's medium supplemented with 10% new born calf serum (Atlanta Biologicals, Flowery Branch, GA) and the antibiotic. Antibodies were purchased from BD (San Jose, CA), Santa Cruz Biotechnology (Santa Cruz, CA), Cell Signaling Technology (Dancers, MA) or Abcam (Cambridge, MA). The *shRNAs* were purchased from Origene (Rockville, MD).

### Active Ras pull down and detection assay

Cell lysates were collected and assayed by the active Ras pull-down and detection kit (Thermo Scientific, Waltham, MA). Briefly, the GTP-form of Ras was pulled down by a GST-fusion protein with the Ras-binding domain (RBD) of Raf attached to glutathione agarose. The pull-down complexes were washed and separated on a 10% SDS-PAGE gel and immunoblotted with an anti-pan-Ras antibody.

### Flow cytometry analysis

DNA fragmentation data were collected by a Muse Cell Analyzer, and analyzed by the Muse software program (BD Biosciences, Franklin Lakes, NJ). Following treatments, cells were harvested and fixed in 70% cold ethanol. Afterwards, cells were stained with 0.1 μg/ml propidium iodide containing 1.5ng/ml RNase. Fragmented DNA contents of cells were then analyzed using a flow cytometer.

### Immunoblotting analysis

Cell lysates were extracted. After DNAs were removed, lysates were run on SDS-PAGE gels. Subsequently, gels were transferred to nitrocellulouses, blocked in TBS containing 5% non-fat milk and probed with corresponding antibodies. Membranes were then visualized by Odyssey infrared imaging system (Li-COR Biosciences, Lincoln, NE).

### ROS analysis

Treated or untreated cells were washed with ice-cold PBS and resuspended in 5 μg/ml of 2′, 7′-dichlorodihydrofluorescein diacetate (DCF) (Thermo Scientific, MA). The samples were incubated for 10 min at room temperature and analyzed immediately.

### Preparation of the cytosolic, cytoplasma membrane and nuclear fractions

Cells were suspended in lysis buffer (200 mM Sucrose, 20mM HEPES, 10 mM KCl, 1.5 M MgCl2, 1 mM EDTA, 1 mM EGTA, 1 mM DTT, Leupeptin, aprotinin at pH 7.4). The lysates then passed through a 25 gauge needle 15 times with syringes. Nuclear pellets were obtained by centrifugation. The remaining supernatants were further centrifuged at 8000 rpm and pellets were removed. The membrane and cytosolic fractions were separated by centrifuging the collected supernatants at 40,000 rpm. Subsequently, the supernatant cytosolic fractions were separated from the pellet plasma membrane fractions.

### Xenograft assay

Cells (1 × 10^6^) in 100 μl of PBS were inoculated into each BalB/c nude mouse. One group of mice (4 mice/group) was injected peritoneally with GO6976 (30 mg/kg) right after the inoculation and subsequently administrated the inhibitor every 3 days. The sizes of the tumors were measured weekly and plotted. After the mice were sacrificed, the tumors were isolated and slides mounted with tumor samples were stained with TUNEL staining. All the animal experiments were carried out according to the guidelines of the Animal Care and Use Committees of the Institute.

### Statistical analysis

Statistical analysis was performed using a two-tailed Student's *t* test for comparison of two groups or a one-way analysis of variance for comparison of more than two groups followed by Tukey's multiple comparison tests. Tumor-free probabilities were estimated using Kaplan-Meier method and were compared among groups. Standard deviations are displayed in the figures. A *p value* < 0.05 was considered significant.
