# Correction: Suppression of PKC causes oncogenic stress for triggering apoptosis in cancer cells

**DOI:** 10.18632/oncotarget.28251

**Published:** 2023-02-25

**Authors:** Suthakar Ganapathy, Bo Peng, Ling Shen, Tianqi Yu, Jean Lafontant, Ping Li, Rui Xiong, Alexandros Makriyannis, Changyan Chen

**Affiliations:** ^1^Center for Drug Discovery, Northeastern University, Boston, MA, USA; ^2^The First Affiliated Hospital, Zhengzhou University, Zhengzhou, China; ^3^The Institute of Clinic Sciences, Sahlgrenska Academy, Gothenburg, Sweden; ^*^These authors contributed equally to this work

**This article has been corrected:** In Figure 3, the panel B image of MIA - MnSOD contains an accidental duplicate of the Figure 6, panel B image of MIA - GADD153. The corrected Figure 3B, produced using the original data, is shown below. The authors declare that these corrections do not change the results or conclusions of this paper.

Original article: Oncotarget. 2017; 8:30992–31002. 30992-31002. https://doi.org/10.18632/oncotarget.16047

**Figure 3:**
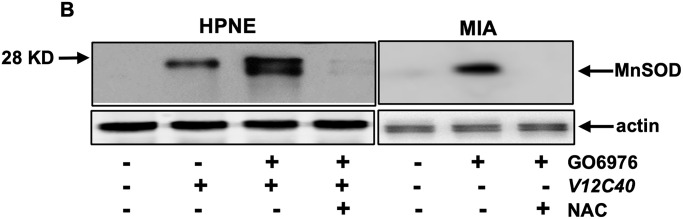
ROS regulators were activated after co-suppression of PKC α and β. (**B**) MnSOD expression in the cells after the treatments was analyzed by immunoblotting.

